# How are adolescents engaged in obesity and chronic disease prevention policy and guideline development? A scoping review

**DOI:** 10.1186/s41256-023-00294-2

**Published:** 2023-03-27

**Authors:** Mariam Mandoh, Julie Redfern, Seema Mihrshahi, Hoi Lun Cheng, Philayrath Phongsavan, Stephanie R. Partridge

**Affiliations:** 1 Faculty of Medicine and Health, School of Health Sciences, The University of Sydney, Westmead, NSW 2145 Australia; 2 The George Institute for Global Health, The University of New South Wales, Camperdown, NSW 2006 Australia; 3 Department of Health Sciences, Macquarie University, Macquarie Park, NSW 2109 Australia; 4 Faculty of Medicine and Health, Sydney Medical School, Specialty of Child and Adolescent Health, The University of Sydney, Westmead, NSW 2145 Australia; 5 Academic Department of Adolescent Medicine, The Children’s Hospital at Westmead, Westmead, NSW 2145 Australia; 6 Prevention Research Collaboration, Faculty of Medicine and Health, Charles Perkins Centre, Sydney School of Public Health, The University of Sydney, Camperdown, NSW 2006 Australia

**Keywords:** Adolescent, Youth, Engagement, Decision-making, Participation, Obesity, Chronic disease, Prevention, Policy, Guideline

## Abstract

**Background:**

Adolescent consumer engagement is widely accepted, with global calls to meaningfully involve adolescents for effective and tailored policy and guideline development. However, it is still unclear if and how adolescents are engaged. The aim of this review was to determine if and how adolescents meaningfully participate in policy and guideline development for obesity and chronic disease prevention.

**Methods:**

A scoping review was conducted guided by the Arksey and O’Malley six stage framework. Official government websites for Australia, Canada, United Kingdom, and United States including intergovernmental organizations (World Health Organisation and United Nations) were examined. Universal databases Tripdatabase and Google advanced search were also searched. Current and published international and national obesity or chronic disease prevention policies, guidelines, strategies, or frameworks that engaged adolescents aged 10–24 years in meaningful decision-making during the development process were included. The Lansdown-UNICEF conceptual framework was used to define mode of participation.

**Results:**

Nine policies and guidelines (n = 5 national, n = 4 international) engaged adolescents in a meaningful capacity, all focused on improving ‘health and well-being’. Demographic characteristics were poorly reported, still most ensured representation from disadvantaged groups. Adolescents were primarily engaged in consultative modes (n = 6), via focus groups and consultation exercises. Predominantly in formative phases e.g., scoping the topic or identifying needs (n = 8) and to a lesser extent in the final stage of policy and guideline development e.g., implementation or dissemination (n = 4). No policy or guideline engaged adolescents in all stages of the policy and guideline development process.

**Conclusion:**

Overall, adolescent engagement in obesity and chronic disease prevention policy and guideline development is consultative and rarely extends throughout the entire development and implementation process.

**Supplementary Information:**

The online version contains supplementary material available at 10.1186/s41256-023-00294-2.

## Background

New and evolving diet and physical activity risk factors such as COVID-19 lockdowns [1], changing food environments and digitalisation and virtual entertainment have made adolescents (10–24 years) more susceptible to obesity and its chronic disease co-morbidities [2–4] compared to previous generations. Increasing evidence is emerging to support consumer engagement in health policy and guideline development to optimise public health solutions for those who they intend to benefit [5]. Consumer engagement is now recognised as a vital component in the development of relevant, effective and evidence informed strategies to tackle public health issues [6, 7].

The importance of engaging adolescents is widely documented, the United Nations (UN) has placed adolescents at the centre of Sustainable Development Goals (SDGs), recognising the importance of engaging young people in decision-making that affects their life and their health [8]. A number of UN and World Health Organisation (WHO) guidelines and guidance documents [9–12] acknowledge the value of and advocate for the inclusion of adolescents in decision-making, design and delivery of interventions. Adolescent participation in chronic disease prevention decision making, affirms not only adolescents potential to improve public health interventions but also the fundamental role adolescents play in social systems, economic growth, and technological development [13, 14].

Similarly, national health strategies have accepted the importance of engaging consumers in obesity and chronic disease prevention policy design and implementation [15, 16]. The United Kingdom’s (UK) National Institute for Health Care Excellence (NICE) guidance [17] and Australian National Health and Medical Research Council (NHMRC) [7] have imbedded consumer engagement within the policy and recommendation development process. Though, not specific to adolescents, the emphasis on engaging relevant consumers from broad walks of life is linked to the development of evidence-based recommendations such as the Australian Dietary Guidelines [18]. Nevertheless, it is still unclear how and to what extent adolescents are engaged in obesity and chronic disease prevention policy and guideline development. The aim of this review is to 1) investigate the frequency of chronic disease prevention policies and guidelines that incorporate adolescent engagement and, 2) to assess the mode and nature of adolescent participation in policies or guidelines developed for chronic disease risk factor reduction, specifically, physical activity, diet, overweight, or obesity.

## Methods

### Study design

The Arksey and O’Malley six-stage framework [19] and Levac et al.’s [20] recommendations were used to guide this review to ensure the vast body of grey literature was thoroughly and iteratively mapped. The review is described based on the Preferred Reporting Items for Systematic reviews and Meta-analysis extension for Scoping Reviews (PRISMA-ScR) checklist (Additional file 1) [21]. The protocol is registered with the Joanna Briggs Institute and Open Science Framework, doi:19.17605/OSF.IO/E3S64, and is published elsewhere [22].

### Eligibility criteria

This review sought to uncover the extent of adolescent participation in policies and guidelines aimed at preventing obesity and chronic lifestyle diseases in adolescents. The review was not intended to provide an assessment of quality of the individual policies or guidelines. The inclusion criteria were defined based on the PCC (Population, Concept, Context) framework as recommended by the Joanna Briggs Institute [23].

#### Population

Adolescents aged 10 to 24 years old [24]. This age range was selected to encompass the range of definitions present within the scientific literature. WHO defines an ‘adolescent’ as a person between the age of 10–19 years,’youth’ as 15–24 years old, and ‘young people’ as individuals between 10 and 24 years [25]. The overlap between age groups has led to the loose and interchangeable use of these terms in the obesity and chronic disease prevention literature. To ensure adequate coverage of adolescent participation in policy and guideline development landscape the study team classified an adolescent as one within age range of 10–24 years old.

#### Concept

The concept under investigation is adolescent participation. Participation was defined based on the Lansdown-UNICEF conceptual framework for measuring outcomes of adolescent participation. A preliminary search determined that the concept of ‘participation’, is often used synonymously with ‘engagement’ and ‘decision-making’ to represent adolescents individually or collectively taking part or involved in influencing activities or matters that impact their life [26].

#### Context

Grey literature was examined in the context of obesity and non-communicable disease prevention. Policies and guidelines related to all aspects of nutrition, diet, healthy eating, and physical activity were extracted and assessed. Policies and guidelines were limited to countries of similar demographic and health ethos and included Australia, Canada, the United Kingdom, and the United States. Policy and guideline documents from the UN and WHO were also examined to better understand the international obesity and chronic disease prevention policy and guideline context. Policy and guideline documents of all languages with a summary published in English were considered for inclusion. Furthermore, our review encompassed policy and guideline documents published from the year 1995–2021. Documents published within this year range, and which were current were included for review as these were determined to be the most relevant in understanding and guiding current practice.

#### Source of evidence

A preliminary search determined the most relevant grey literature sources for the purpose of this review. It was decided that policy, guideline, strategy, and framework documents would be included in the review as they play a significant role in the context of national public health agendas. Definitions are outlined in Table 1. In this review we will refer to policy, guideline, strategy, and framework documents collectively as ‘policy and guideline documents.’

**Table 1:** Definitions

Term	Definition
Policy	A high-level course or method of action to guide and determine present and future decisions [27]
Guideline	An indication or outline of policy or conduct [28]
Strategy	A careful plan or method [29]
Framework	A basic conceptional structure of ideas [30]

The phrase ‘policy and guideline documents’ encompasses the breadth of significance of policy, guideline, strategy, and framework documents with the common goal to influence and form the foundation for national public health agendas and best practice in health delivery [31].

Policies and guidelines that specifically target adolescents and those intended for the general population were considered for inclusion. Policies and guidelines in which all participants were aged at either extreme of the adolescent age bracket ‘10–12’ years or ‘20–24’ years were excluded. The rationale for this decision was based on the awareness that pre-teens (10–12 years) and young adults (20–24 years) differ significantly in their lived experience, as well as biological and psychosocial development and therefore participation for each of these groups would have a different meaning [32]. A representative sample of adolescents would ideally comprise adolescents of varied ages.

### Information sources

National and international grey literature information sources were investigated between January 2021 and February 2022, inclusive. National websites of policy and guideline databases, as well as official organization websites of the UN and WHO were examined (Table 2). Next, a grey literature register (TRIP database) and a custom search engine (Google advanced search) were examined for additional policy and guideline documents associated with the countries and international organizations of interest.

**Table 2:** Information sources

Country/international organization	Grey literature sources
Australia	National Health and Medical Research Council [33] Department of Health and Aged Care [34]
Canada	Health Canada [35]
The United Kingdom (UK)	National Institute for Health and Care Excellence [36]
The United States (US)	Agency for Healthcare Research and Quality [37]
International organizations	United Nations (UN) [38] United Nations Children’s Fund (UNICEF) [39] World Health Organization (WHO) [40]
All (Aust, Canada, UK, US, UN, WHO)	Google Advanced search [41] Tripdatabase.com [42]

### Search

Search terms reflecting the PCC concepts and source of evidence were developed by the research team in consultation with an academic liaison librarian. Terms included ‘policy’, ‘framework’, ‘adolescent’, ‘youth’, ‘teenager’, ‘young people’, ‘nutrition’, ‘health’, ‘physical activity’, ‘obesity, ‘prevention’, ‘decision-making’, ‘participation’ and ‘engagement’. Phrases identified encompassed ‘chronic disease prevention’, ‘obesity prevention’, ‘obesity prevention in youth’, ‘obesity prevention framework’, ‘overweight and adolescence’, ‘physical activity guidelines’, ‘diet and young people’, ‘youth health’, ‘adolescent nutrition’, ‘nutrition guidelines’ ‘nutrition policy’ and ‘youth health policy’. Terms and phrases were searched across country and organization specific sites. It was agreed that searches would be limited to the first five pages, or first 50 records of results as they would comprise the most relevant records. The search strategy varied slightly between websites depending on the search function capabilities. Advanced search options were used when available. Where only the basic search function was present, key words and phrases were searched followed by a thorough hand search of the site.

### Selection of sources of evidence

**Fig. 1:**
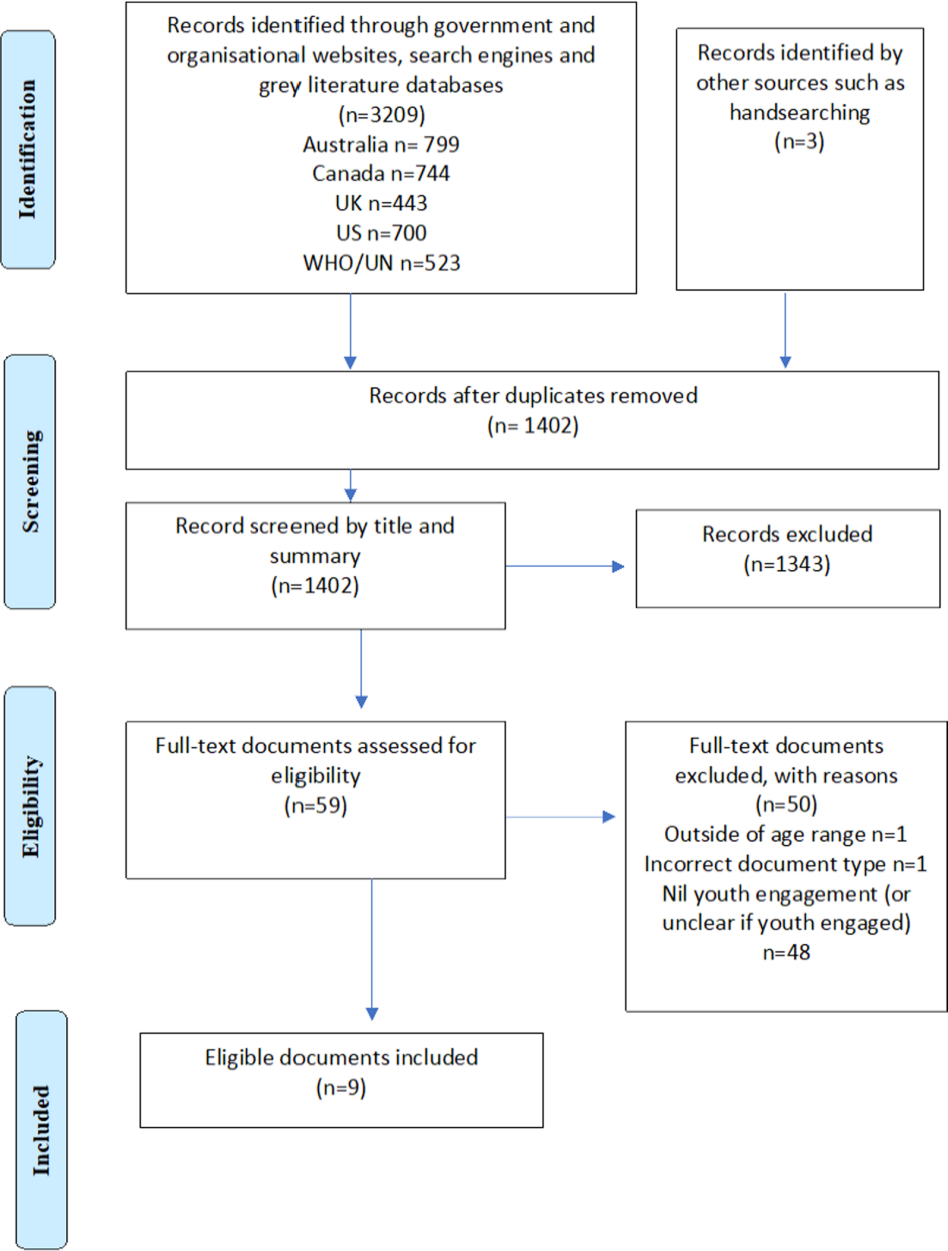
Prisma flow diagram: adolescent engagement in policy and guideline development for obeity and chronic disease prevention

Initial screening of title and summary or table of contents by reviewer one (MM) was undertaken to determine relevance of the document. Duplicates were removed and review of the full text of the remaining documents followed. Full-text screening was undertaken by reviewer one (MM) and checked by reviewer two (SRP) for agreement (Fig. 1).

### Data charting, data items and synthesis

As per scoping review guidelines a data extraction form was developed in Excel and piloted by the research team to ensure the necessary data were obtained [23]. Data extraction was conducted by one reviewer (MM) and checked by another reviewer (SRP) for confirmation and agreement. Data charting categories were developed to reflect characteristics of sources of evidence, demographic data, purpose of adolescent engagement, main chronic disease or risk factors of interest and the mode and nature of adolescent participation in the policy development process.

#### Mode of adolescent participation

The Lansdown-UNICEF conceptual framework for measuring outcomes of adolescent participation [26] informed data charting categories representing the mode of adolescent participation. The mode of participation was defined based on the degree of influence adolescents imparted on the policy and guideline development process. ‘Consultative’ participation, involves adults leading and managing, while adolescents are given the opportunity to express their views. ‘Collaborative’ participation typifies a partnership between the adults and the adolescents, with adolescents having more influence over the process and the outcomes. ‘Adolescent-led’ participation represents the highest degree of influence, where adults act as facilitators, while adolescents have full control over the process and the outcomes.

#### Nature of adolescent engagement

The Australian NHMRC consumer involvement guidelines [7] informed data categories representing the nature of adolescent engagement in the policy and guideline development process. The phases of adolescent engagement were defined as ‘Stage 1. ‘Scoping the topic and Identifying needs’ to ensure policies are relevant to the consumer. ‘Stage 2. Planning’, to ensure outcomes of most importance are addressed. ‘Stage 3. Conducting an evidence review’, to incorporate existing evidence into the process. ‘Stage 4. Reviewing draft recommendations’, to ensure policies are practical and suitable for the target consumer group. ‘Stage 5. Resource development’, to ensure that associated resources are suitable for the target group and, ‘Stage 6. Implementation/ dissemination’, to enable adolescent consumers to use their experience and knowledge to guide the policy implementation and dissemination process.

Participatory outcomes were assessed based on the Lansdown-UNICEF conceptual framework [26]. Data charting categories reflected participatory markers of empowerment and influence including, ‘sense of self-worth/ self-esteem/ efficacy’, ‘being taken seriously’, ‘making decisions’ and ‘public/civic engagement’. Obesity and chronic disease outcomes were not charted as these data were not available in the policy and guideline literature. Qualitative data were collected in the data extraction form by reviewer one (MM) and checked by reviewer two (SRP) for consistency.

## Results

A total of five national government department websites [33–37], three international sites [38–40] and two universal databases [41, 42] were searched. Overall, 1402 records were screened by title and summary (Fig. 1). Next, 59 full-text policy and guideline documents were screened for eligibility. Of the full-text documents reviewed, 50 documents were excluded with reasons (Fig. 1) and nine policy and guideline documents met all inclusion criteria and were examined within this review.

### Characteristics of sources of evidence

Table 3 summarises the characteristics of each policy and guideline document. The nine grey literature documents included comprised of three guidelines [11, 43, 44], two strategies [16, 45], three frameworks [46–48], and one policy [49]. All policy and guideline documents were published from 2015 onwards, with two published in 2021. Overall, four documents were under international jurisdiction, published by the WHO alone or in partnership with UN agencies with the aim to influence policy and intervention development of national policymakers. Two WHO policy documents were linked, one was a global health strategy [10] and the other its implementation guidance specifically targeting an adolescent audience [11]. Five policy documents were published under national jurisdiction, two Australian [43, 46], two Canadian [16, 49] and one British [47].

**Table 3:** Adolescent participation in policy and guideline development data extraction

References	Document name	Document type/policy format	Country/organisation	Jurisdiction	Demographics (adolescent)	Purpose of engaging adolescents	Duration of participation/participatory activities conducted	Adolescent engagement description	Main chronic disease or risk factors of interest
Public Health England [47]	Improving young people’s health and wellbeing: A framework for public health	Framework	Public health England	National	N = 51 (10–24 years)—survey NR for discussions Mixed group, included vulnerable adolescents	To include adolescent voices/ opinions into policy and program development	NR	Surveys and twitter ‘discussion hours’	Health and well-being
WHO [10]	Global Strategy for women’s children’s and adolescent’s health (2016–2030)	Strategy	WHO	International	NR	Variety of views/stakeholder representation	12 months	Face-to-face consultations, via conferences, surveys	Health and well-being
Health Canada [16]	Health Canada’s Healthy Eating strategy	Strategy	Canada, Health Canada	National	N = 97 (17–24 years) N = 2 (< 17 years) General members of the public	Variety of views/ stakeholder representation	2 years	Online consultation workbook	Health and wellbeing- healthy eating
WHO [11]	Global Accelerated Action for the Health of Adolescents (AA-HA!): guidance to support country implementation	Guideline	WHO	International	NR Young and vulnerable adolescents included	“Nothing for them without them’, “To put young people in the driver’s seat”	NR	Consultation workshops Helped develop adolescent friendly version of guidance	Health and well-being
Government of Canada [49]	Canada’s Youth Policy	Policy	Canadian government	National	Online have your day booklet participants Total N = 4804 N = 1690 (< 16 years old) N = 1512 (17–20 years old) N = 1601 (21–24 years old)	To give adolescents a voice in the decision making	NR	Online ‘have your say’ booklet Online discussion forum Video comment submissions Youth roundtables face-to-face (discussions)	Physical and mental health
Commonwealth Department of Health [43]	Australian 24-h movement guidelines for children (5–12 years) and young people (13–17 years): an integration of physical activity, sedentary behaviour, and sleep	Guideline	Australia, Department of Health	National	N = 10 (9–16 years) Include indigenous adolescents & mixed backgrounds	To ensure adolescents will be able to use the resource	NR	Reviewed draft resource, interviews, focus groups, dissemination strategies discussion	Health and wellbeing
Ross et al. [48]	Adolescents Well-being framework	Framework	UN H6+	International	Online consultations N = > 340 ((13–29 years) Other activities NR Mixed group, Included indigenous and vulnerable adolescents	To include adolescent voices/ opinions into policy and program development	NR	Consultations Involvement in planning of the global summit for adolescent well-being 2023	Health and well-being
UNESCO [44]	Making every school a health-promoting school: Implementation guidance	Guideline	WHO/ UNESCO	International	NR	To ensure adolescents will be able to use the resource	NR	Consultations	Health and well-being
Department of education, skills and employment [46]	Australia’s Youth Policy framework	Framework	Australian government	National	15–24 years Focus on marginalised adolescents	To include adolescent voices/ opinions into policy and program development	> 2 months	Consultation exercises Surveys	Health and wellbeing

*NR* not reported; *Y* yes

### Demographics

Demographic characteristics of adolescent participants were ambiguously reported in all nine policy and guideline documents examined. Gender of participating adolescents was the most overlooked characteristic and was not reported in any of the documents reviewed. Age range was reported in six of the nine policy and guideline documents, with wide variations in age stratification of adolescent participants across the documents [16, 43, 46–49]. Age groups included ‘ < 16 years’, ‘ < 17 years’, ‘9–16 years’, ‘10–24 years’, ‘15–24 years’, ‘17–20 years’, ‘17–24 years’, ‘13–29 years’ and ‘21–24 years’ (the older age group was only included if the policy or guideline development process also included younger adolescent age groups).

The number of participants reported varied significantly between policy and guideline documents. The lowest number of participants was ten [43] and the highest number was 4804 [49]. Overall, international documents reported on demographic characteristics of participating adolescents the least [10, 11, 44, 48]. All four international documents failed to report any details on age or number of participating adolescents. Five of the nine documents reported an intentional focus on including first nations, vulnerable, or marginalised adolescents [11, 43, 46–48].

### The purpose of engaging adolescents

The reasons for engaging adolescents in the policy and guideline development process varied broadly between policy and guideline documents (Table 3). Two guidelines engaged adolescents to ensure that these resources could practically be utilised by adolescents themselves [43, 44]. Four documents sought to ensure that adolescent voices and concerns were addressed and incorporated into future program and intervention development [46–49]. Engagement motives also included incorporating a variety of stakeholder views and representation [16, 45] or to give adolescents more control over the decision-making process and “to put young people in the driver’s seat” [11].

### Main chronic disease or risk factors of interest

Improving health and wellbeing was the main focus of all nine of the policy and guideline documents examined (Table 3), though ‘Canada’s Health Policy’ also included mental health within its scope [49]. The documents under examination varied in their approach to improving health and wellbeing. Two policy and guideline documents targeted specific chronic disease risk factors, either through promoting physical activity [43] or improving eating habits [16], while others had a more broad focus and endeavoured to take into consideration all facets of adolescent health and determinants of health for young people [10, 11, 44, 46–49].

### Participation

#### Mode and nature of adolescent participation

The mode in which adolescents participated in the policy and guideline development process was relatively consistent across nine policy and guideline documents reviewed (Table 4). Six of the nine policy and guideline documents involved adolescents in a consultative capacity, while one implementation guidance [11], one policy [49] and one framework [48], engaged adolescents in a collaborative mode. Collaborative participation was intended by policymakers to give adolescents more power over the decision-making process, still this only correlated to involvement in 30% [11, 49] or 50% [48] of stages of the policy and guideline development process.

**Table 4:** Mode and nature of adolescent participation in policy and guideline development

References	Document name	Mode of adolescent participation (Lansdown-UNICEF conceptual framework)			Nature of adolescent engagement in the policy/guideline development process (Australian NHMRC consumer involvement guidelines)						Participatory outcomes (Lansdown-UNICEF conceptual framework)			
		Consultative	Collaborative	Adolescent-led	1. Scoping the topic/ identifying needs	2. Planning	3. Conducting evidence review	4. Reviewing draft recommendations	5. Resource development	6. Implementation/ dissemination	Sense of self-worth/self-esteem/efficacy	Being taken seriously	Making decisions	Public/ civic engagement
Public Health England [47]	Improving young people’s health and wellbeing: A framework for public health	Y			Y						NR	NR	NR	NR
WHO [10]	Global Strategy for women’s children’s and adolescent’s health (2016–2030)	Y			Y						NR	NR	NR	NR
Health Canada [16]	Health Canada’s Healthy Eating strategy	Y			Y						NR	NR	NR	NR
WHO [11]	Global Accelerated Action for the Health of Adolescents (AA-HA!): guidance to support country implementation		Y		Y				Y		NR	NR	NR	NR
Government of Canada [49]	Canada’s Youth Policy		Y		Y					Y	NR*	NR*	NR*	NR*
Commonwealth Department of Health [43]	Australian 24-h movement guidelines for children (5–12 years) and young people (13 to 17 years): an integration of physical activity, sedentary behaviour, and sleep	Y						Y		Y	NR	NR	NR	NR
Ross et al. [48]	Adolescent Well-being framework		Y		Y	Y				Y	NR*	NR*	NR*	NR*
UNESCO [44]	Making every school a health-promoting school: Implementation guidance	Y			Y						NR	NR	NR	NR
Department of Education, Skills and Employment [46]	Australia’s Youth Policy framework	Y			Y	Y				Y	NR	NR	NR	NR

*NR* not reported; *Y* yes

*Participatory outcomes were not measured or reported; however, participants were asked about the importance of these outcomes, and these were discussed during the participatory exercises

Overall, all nine policy and guideline documents involved adolescent consumers in at least one of the six stages of the policy and guideline development process. Five documents involved adolescents in more than one stage, with three policy and guideline documents engaging youth in two stages and two engaging youth in three stages [46, 48]. The majority (8/9) of policy and guideline developers engaged adolescents in stage 1 of the policy and guideline development process ‘scoping the topic and identifying needs’. Two policy and guideline developers engaged young people in the ‘planning’ stage although this was within a consultative capacity [46] [48]. Another engaged youth in ‘reviewing draft recommendations’ [43] to ensure useability by adolescents themselves. In the development of an additional guideline adolescent consumers were engaged in ‘resource development’ assisting in the development of an adolescent friendly version of the guidance. Four policy and guideline documents reported young people informed ‘implementation or dissemination’ strategies such as providing recommendations for action [43, 46, 49] and involvement in the planning of a global conference to advocate for framework implementation [48].

#### Participatory methods

All nine policy documents included within this review utilised consultation, discussion or focus group exercises to engage adolescents in the decision-making process (Table 3). Still, consultation exercises varied in approach, four policy and guideline developers employed online/digital platforms [16, 47–49], one specifically reported consulting with young people face-to-face [10, 49] and four did not specify consultation approach [11, 43, 44, 46]. Additionally, surveys were used in the development of three policy and guideline documents [10, 46, 47], conference meetings in one global strategy [10], video comment submissions in another [49] and interviews in one national guideline [43]. With a maximum of four participatory methods used in the development of a policy or guideline.

#### Participatory outcomes

All nine policy and guideline documents reviewed indicated engaging adolescents as a priority. However, participatory guiding principles such as participatory action research strategies were only recommended in one document [44]. Furthermore, participatory outcomes were not measured or reported in any of the policy and guideline documents reviewed (Table 4); therefore, participatory outcomes could not be examined in this review. Nevertheless, one policy document acknowledged the importance of participatory outcomes by asking adolescent participants about their opinions on participatory outcomes such as civic engagement [49].

## Discussion

In this review we examined the emerging concept of adolescent participation in the development of national and international obesity and chronic disease prevention agendas. All the policies and guidelines included within this review were published in the last seven years, with health and well-being the core focus. International and national public health agendas emphasise the significance of adolescent consumer engagement on matters relevant to adolescents [8, 16, 50, 51]. This message is reiterated by policymakers and funding bodies as a recommendation for best practice and critical to the attainment of sustainable development goal targets [7, 11, 52]. Our review revealed that current recommendations for adolescent consumer engagement are rarely reflected in the obesity and chronic disease prevention policy and guideline development process. Yet, our review also indicates that international organisations are more committed to engaging adolescents compared to national governments, though outcomes of such efforts are yet to be seen [53].

It is apparent that policies and guidelines that currently involve some forms of adolescent engagement have focussed mainly on general health and wellbeing rather than specific health risk factors like obesity. For example, Australia and Canada’s national youth policies were developed in consultation with adolescents and exemplify a whole of systems approach to adolescent engagement [46, 49]. While a general health and wellbeing focus is important in recognising the multitude of biopsychosocial determinants of chronic disease [54] ensuring adequate adolescent engagement for obesity policies is equally important in order to generate effective solutions for this pressing public health issue [8]. Nevertheless, only nine policy and guideline documents met inclusion criteria for this review, indicating that the application of youth engagement in policy and guideline development for chronic disease prevention is infrequent and is still an objective and unregulated practice.

Overall, this review revealed systematic inconsistencies in the reporting of demographic characteristics as well as reporting on the nature of adolescent involvement in obesity and chronic disease prevention policy and guideline development. This meant that several polices, and guidelines could not be included in this review. Inconsistent reporting creates challenges for establishing the incidence and manifestation of adolescent engagement in obesity and chronic disease prevention policy and guideline development [9, 17]. Incomplete reporting of adolescent engagement data in different data domains was reflected in the UN Youth 2030 strategy [9]. The UN Youth 2030 strategy was developed to enhance efforts to achieve the SDGs by meaningfully engaging youth in the implementation of the strategy. Youth are engaged to varying degrees in the national and international implementation of components of the strategy, albeit incomplete data on ‘youth’ participants, it is unclear which SDGs were targeted and whether youth engagement played any part in SDGs related to improving health and reducing the burden of chronic disease [55, 56]. Furthermore, the UK’s NICE guidelines for obesity and chronic disease prevention [17, 57] and Australia’s Preventative Health Strategy [15] recognise the importance of engaging consumers in the policy and guideline development process. However, these documents make reference to consultation with ‘people using the health and care services’, ‘people from communities affected by the guideline’ [58] or ‘consumer or community engagement’ [15] without providing context or detail of their ages. Conversely, Australia’s National Obesity Strategy 2022–2032 explicitly engaged ‘young people’ in ‘targeted engagement’. Although their consultation with 21–26-year old’s was not representative and does not truly address the needs of the general adolescent population [59]. Poor data reporting, low participant numbers [43, 59, 60] and inclusion of only ‘young adult’ youth [59] deny adolescents equitable representation on matters that are important to them.

Unclear reporting of participant data is not unique to the adolescent engagement literature and is apparent across the obesity and chronic disease prevention policy and guideline literature [52, 61]. On one hand, these current practices may be viewed as tokenistic and may be attributed to the increasing requirement to engage consumers as part of ‘best practice’ guidelines for policymakers [6, 62] and a lack of a universal adolescent engagement framework. On the other hand, such practices may also be the first step toward greater participation of adolescents in policy development as nations recognize the importance of end user engagement in policy making. Despite the emphasis on consumer engagement in policies and guidelines developed for adolescents [63, 64], adolescents are yet to be recognised as fundamental stakeholders despite their unique insight which many policy and guideline development processes require [65]. Furthermore, our review determined that when engaged within obesity and chronic disease prevention policy and guideline development processes, participation is superficial in nature. A consultative capacity was the primary mode of participation, while no policy or guideline documents assessed within this review engaged adolescents in an adolescent-led approach. This is consistent with findings from adolescent engagement in health research literature [66].

Adolescents are more likely to be involved in the formative stages of the policy and guideline development process, where perspectives and needs are established. Not involving adolescents throughout the entirety of the policy and guideline development process has implications for policy and guideline design, translation, and implementation. In grassroots initiatives at state and local levels this has resulted in inconsistencies in outcomes of adolescent engagement [67]. Increasing popularity of youth councils, youth parliaments and youth advisory groups at local and community levels has resulted in pockets of action with no national consensus [6, 11, 67]. A consolidated effort is necessary to ensure adolescent engagement is meaningful and impactful [51].

Furthermore, participatory outcome data in the policy and guideline literature is lacking therefore it is unclear how participation is impacting adolescent consumers. Participatory outcomes such as empowerment and influence enable adolescents to help themselves and their peers to improve their own lives and reduce chronic disease risk factors [51]. Yet this review has found that policies and guidelines aimed at improving health and wellbeing of adolescents are not underpinned by participatory principles or frameworks.

This scoping review has several limitations which were challenging to mitigate, however important to note. The review was limited to established chronic disease risk factors, namely diet, physical activity and overweight and obesity. Other risk factors, such as sleep, and screen time were outside the scope of this review and were not included as individual search terms. An absence of standardised measures to quantify participatory outcomes, meant that measurement and documentation of participation was subjective in nature. Within the literature the term ‘participation’ is often used to refer to participants taking part in a process, however not necessarily engaged in a meaningful manner as per participatory frameworks [26]. Furthermore, within the grey literature the use of ‘consumer’ or ‘stakeholder’ consultation was often used. However, a paucity of specific details on the participants of the consultations, made it difficult to determine the extent and nature of adolescent engagement. Additionally, this review was limited to documents that reported adolescent engagement in policy and guideline development. Recognising that there may potentially be policies or guidelines which engaged adolescents however did not report it within the published policy or guideline documents. Moreover, documents that were published after the end of the systematic search may have been missed. Finally, this review was limited to policy and guideline documents of the specified high-income countries and organisations. Therefore, this review does not reflect the state of adolescent participation in policy and guideline development in Low- and Middle-Income Countries (LMICs) or globally.

A limitation specific to reviewing the grey literature included the fact that different sites and databases have different search options and navigation tools making a standardised search strategy challenging to execute. Scoping reviews have innate limitations of importance to consider. By design scoping reviews are broad in scope and aim to map the literature therefore the included policy and guideline documents were varied in their scope and purpose. This can make direct comparisons between the policy and guideline documents challenging. Furthermore, although search terms used were broad and the strategy was systematic, as with any review it is possible for some policy and guideline documents to have been missed. Moreover, policy and guideline documents were limited to those published in English, this self-selection limitation may have by default excluded documents not published in English. Further, the policy and guideline search were limited to countries with similar demographics and health ethos and would therefore not be representative of the entire global condition. Finally, scoping review guidelines, deem it unnecessary to rate the quality of the data or conduct a critical appraisal of the evidence used in scoping reviews, this may have implications for practice [23].

## Conclusions

Adolescent consumer engagement is recognised as a component of best practice for chronic disease prevention policy and guideline development yet appears to be scarcely implemented. Furthermore, participatory frameworks need to address small participant numbers and inconsistencies in reporting to enhance representation, translation, and transparency of adolescent participation in chronic disease prevention policy and guideline development.

## Supplementary Information

**Additional file 1**. Preferred Reporting Items for Systematic reviews and Meta-Analyses extension for Scoping Reviews (PRISMA-ScR) checklist.

## Data Availability

All data generated or analysed during this study are included in this published article [and its Additional file 1].

## Abbreviations

UN: United Nations
SDGs: Sustainable Development Goals
WHO: World Health Organization
UK: United Kingdom
NICE: National Institute for Health Care Excellence
NHMRC: National Health and Medical Research Council
PRISMA-ScR: Preferred Reporting Items for Systematic reviews and Meta-analysis extension for Scoping Reviews
PCC: Population, Concept, Context
UNICEF: United Nations International Children’s Emergency Fund
UNESCO: The United Nations Educational, Scientific and Cultural Organization
UN H6+: A collaboration between UNFPA, UNICEF, UN Women, WHO, UNAIDS and World Bank Group
AA-HA!: Accelerated Action for the Health of Adolescents
PMNCH: Partnership for Maternal, Newborn and Child Health
UNAIDS: The joint United Nations Programme on HIV/AIDS
UNFPA: United Nations Population Fund
LMICs: Low- and Middle-Income Countries
